# UNDERSTANDING RACIAL AND ETHNIC DISPARITIES IN NURSING HOME CARE IN COOK COUNTY, IL

**DOI:** 10.1093/geroni/igac059.593

**Published:** 2022-12-20

**Authors:** Selena Caldera

**Affiliations:** AARP, Washington, District of Columbia, United States

## Abstract

Nursing facility data from the Illinois Department of Public Health reveals significant racial disparities in access to high quality nursing homes (NH) for older Black and Latino Illinoisans. While half of all Illinois NH residents live in a 1- or 2- star rated nursing home, 68% of Black NH residents live in such facilities. This study seeks to understand racial and ethnic disparities in access to, quality of, and experiences with care in Cook County, Illinois NHs and develop community-identified solutions to close quality, access, and equity gaps. We employ key informant interviews in a two-stage process that begins by developing a current state analysis of the experience with care through interviews with community stakeholders, including advocacy groups, policy and community leaders and public agencies. Those findings then guide interviews with older Black, Latino, and Chinese NH residents and their caregivers where we identify community-grounded solutions to closing equity gaps.

